# Exposure to maternal obesity alters gene expression in the preimplantation ovine conceptus

**DOI:** 10.1186/s12864-018-5120-0

**Published:** 2018-10-11

**Authors:** Sarah R. McCoski, McCauley T. Vailes, Connor E. Owens, Rebecca R. Cockrum, Alan D. Ealy

**Affiliations:** 1 Department of Animal and Poultry Sciences, Virginia Polytechnic Institute and State University, 3430 Litton-Reaves Hall (0306), Virginia, Blacksburg, VA 24061 USA; 2 Department of Dairy Science, Virginia Polytechnic Institute and State University, Blacksburg, VA 24061 USA

**Keywords:** Maternal obesity, Preimplantation, Conceptus, Placenta

## Abstract

**Background:**

Embryonic and fetal exposure to maternal obesity causes several maladaptive morphological and epigenetic changes in exposed offspring. The timing of these events is unclear, but changes can be observed even after a short exposure to maternal obesity around the time of conception. The hypothesis of this work is that maternal obesity influences the ovine preimplantation conceptus early in pregnancy, and this exposure will affect gene expression in embryonic and extraembryonic tissues.

**Results:**

Obese and lean ewe groups were established by overfeeding or normal feeding, respectively. Ewes were then bred to genetically similar rams. Conceptuses were collected at day 14 of gestation. Morphological assessments were made, conceptuses were sexed by genomic PCR analysis, and samples underwent RNA-sequencing analysis. While no obvious morphological differences existed between conceptuses, differentially expressed genes (≥ 2-fold; ≥ 0.2 RPKM; ≤ 0.05 FDR) were detected based on maternal obesity exposure (*n* = 21). Also, differential effects of maternal obesity were noted on each conceptus sex (*n* = 347). A large portion of differentially expressed genes were associated with embryogenesis and placental development.

**Conclusions:**

Findings reveal that the preimplantation ovine conceptus genome responds to maternal obesity in a sex-dependent manner. The sexual dimorphism in response to the maternal environment coupled with changes in placental gene expression may explain aberrations in phenotype observed in offspring derived from obese females.

**Electronic supplementary material:**

The online version of this article (10.1186/s12864-018-5120-0) contains supplementary material, which is available to authorized users.

## Background

Obesity is a prominent cause of various adverse health conditions, including heart disease, stroke, type 2 diabetes, and some cancers in humans and other mammals [1]. The prevalence of these conditions may be one of the leading causes of preventable death among adults. Lifestyle choices and poor diet are recognized as the main factors leading to obesity, however, more recent evidence suggests that intrauterine exposure to an obesogenic environment is a contributing factor predisposing offspring to obesity-related disorders. Approximately one-third of child-bearing age women in the United States (20 to 39 years of age) are overweight, and another one-third are obese [2]. Postnatal eating and dietary habits of offspring increase the likelihood of childhood and adult obesity in offspring, however, obesity-related disorders can manifest in these offspring even in the absence of the obese phenotype [3, 4]. The postnatal onset of these events that were manifested in utero is a hallmark feature of the fetal origins of adult disease (FOAD) or developmental origins of health and disease (DOHAD) phenomena that have been observed in all mammals studied to date. [5]. These adverse outcomes may be caused by epigenetic modifications to the genome or by direct, non-genomic modification of organ and tissue development during the embryonic and fetal periods of gestation.

The initial concept of DOAHD applied to human offspring exposed to under nutrition in utero, however, it has since grown to also encompass the state of over nutrition during early development. Animal models have been used extensively to study this phenomenon. Reports in rodents reveal that increased maternal adiposity results in insulin resistance, hyperlipidemia, and increased body weight in offspring [6]. Additionally, exposure to maternal obesity is linked to altered skeletal muscle function [7] and reduced muscle mass in male and female offspring [8]. The relationship between nutrition in utero and muscle growth is important in animal agriculture, as skeletal muscle development is directly related to meat quality in various species including the sheep [9–11]. Furthermore, ewes subjected to fetal exposure to maternal obesity were hyperglycemic, hyperinsulinemic, and showed significant increases in pancreatic weight at mid-gestation [12]. Similar to the mouse model, the obese ewe produces lambs exhibiting altered growth, adiposity, and glucose tolerance in adulthood [13]. While the effects of maternal obesity are known to have lasting effects in offspring, methods to alleviate these effects are lacking.

The placenta is a prime target for intrauterine stresses, and modifications in placental development and function are linked to several adverse health events that occur after birth [14, 15]. Maternal obesity has a direct effect on placental nutrient transport, placental vasculature, and blood flow [16–19], and interestingly, exposure to maternal obesity alters placental development in a sexually dimorphic manner [20–25]. Similarly, several fetal outcomes observed in offspring exposed to maternal obesity are sexually-dependent, including glucose intolerance, adiposity, blood pressure, and insulin sensitivity [26–28]. The mechanism and timing of the sex-dependent changes in placentation and fetal outcomes are not understood, thus genes involved in placentation were of particular interest in assessing the effects of maternal obesity on the developing embryo.

We were interested in understanding how maternal obesity impacts pre- and peri-implantation embryogenesis. This is a time of significant embryonic and extraembryonic tissue development and cellular restructuring in the embryo and placenta [29]. Critical events occurring during this time include highly controlled changes in embryonic DNA methylation patterns, embryonic cell lineage specification, and embryonic-maternal cross-talk that controls pregnancy recognition [30]. Furthermore, studies utilizing an ovine embryo transfer model showed that exposure to maternal obesity only during the periconceptional period was sufficient to impose altered developmental outcomes in lambs [31, 32]. However, the immediate effects of maternal obesity on conceptus growth and function during peri-implantation development remained unexplored to this point. We propose that exposure to environmental stressors and the resulting disruptions in the genes associated with developmental processes will adversely affect early placentation events and thereby adversely affect embryo competency. The following work examined the validity of this premise by examining the effects of obesity status on reproductive performance and conceptus gene expression profiles of ewes at day 14 of pregnancy.

## Results

### An increased plane of nutrition affects body parameters of ewes

Providing a corn-based diet altered body conformation of ewes (Table 1). Obese ewes had a greater average body weight at the time of collection compared to lean ewes (*P* < 0.0001). Similarly, obese ewes had greater BCS (P < 0.0001) and greater back fat measurements (*P* = 0.002) than their lean counterparts. Obesity did not affect plasma NEFA concentrations, however, NEFA concentrations were reduced at day 14 in both groups (*P* = 0.03) (Table 1). Circulating glucose concentrations were unaffected by obesity status.

**Table 1:** Body parameters of obese and lean ewes

Parameter^c^	Obese	Lean
Number of ewes	13	14
Weight at D14 (kg)	100.6 ± 3.7^A^	64.9 ± 2.4^B^
BCS	4.4 ± 0.1^A^	2.7 ± 0.1^B^
Back fat (cm)	1.5 ± 0.1^A^	0.4 ± 0.1^B^
Plasma glucose (mg/dL)	3.47 ± 0.3	2.49 ± 0.7
Plasma NEFA (mEq/L)		
D0^a^	0.219 ± 0.11	0.250 ± 0.06
D6^a^	0.059 ± 0.03	0.087 ± 0.02
D14^b^	0.037 ± 0.003	0.037 ± 0.003

^a,b^Lower case superscripts indicated differences by day of sampling (*P* < 0.05)

^c^Uppercase superscripts denote differences between groups (*P* < 0.05)

### Obesity does not Alter various pregnancy parameters

Ewes were sacrificed at day 14 post-breeding, and data were collected to assess the effects of obesity on various pregnancy parameters (Table 2). Pregnancy rate, ovulation rate (CL number), and conceptuses/CL (pregnancies/ovulation) were not affected by obesity status. Also, conceptus length, conceptus sex ratio and IFNT production were not affected by maternal obesity status or conceptus sex at day14. Maternal obesity status also had no effect on circulating P4 concentrations at days 0, 6 and 14 post-estrus.

**Table 2:** Ewe pregnancy parameters

Parameter	Obese	Lean
Pregnancy rate (%)	62.5	68.4
CL number	2.07 ± 0.15	1.84 ± 0.16
Conceptuses/CL (%)	96.2 ± 0.05	85.2 ± 0.07
Male:female ratio^a^	6:9 (40:60)	7:6 (54:46)
Mean conceptus length (cm)		
Male	10.46 ± 1.9	8.18 ± 1.8
Female	8.07 ± 1.8	9.57 ± 1.9
Range in conceptus length (cm)	4–26	3–24
Total uterine IFNT (mg)	12.0 ± 4.2	8.1 ± 5.4
Conceptus size-adjusted IFNT (mg/cm)^b^	0.9 ± 0.3	0.6 ± 0.2
Male:female ratio^b^	6:9 (40:60)	7:6 (54:46)
P4 concentration (ng/ml)		
D0	0.5 ± 0.3	0.4 ± 0.3
D6	2.6 ± 0.3	2.9 ± 0.3
D14	4.0 ± 0.3	4.7 ± 0.3

^a^Data presented numerically and as a percentage of total conceptuses within treatment in parentheses.

^b^The IFNT content was adjusted based on the combined size of all conceptuses collected from the flush (total IFNT / cm of all conceptuses in the flush).

### Exposure to maternal obesity affects conceptus gene expression

RNA-sequencing was completed on a subset of samples (*n* = 4 of each sex for obese and lean groups) to assess the effects of maternal obesity exposure on gene transcription in the preimplantation ovine conceptus. There was a concern with the completeness of annotation in the ovine genome assembly, so an initial set of annotations were completed against the ovine, bovine, and caprine genomes. Percentages of reads mapped to each genome were similar among species; with 94.2%, 92.1%, and 94.4% mapped reads when using the ovine, bovine, and caprine genomes, respectively. Thus, the ovine genome annotation was used for the various analyses. The ovine genome identified an average of 32,220,571 reads/sample, 42,390 transcripts/sample (see Additional file 1) and 28,381 genes/sample.

**Fig. 1:**
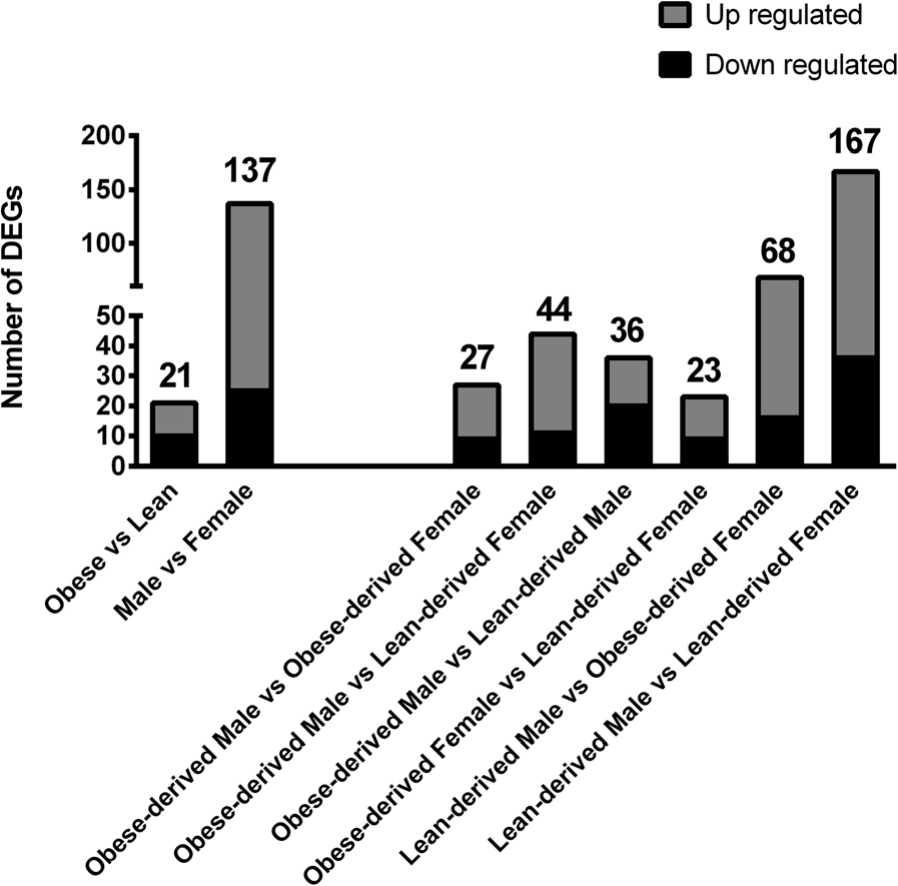
The number of up- and down- regulated genes across experimental comparisons. Ovine conceptuses were collected from obese and lean ewes on day 14 of pregnancy via uterine flush. Conceptus sex was determined by PCR using X- and Y-specific primers, and 4 conceptuses/sex/treatment underwent RNA sequencing (*N* = 16 total conceptuses). Sequencing analysis was performed using Genomics Workbench 10.1.1 (CLC bio). Sequences were mapped to the *Ovis aries* genome (NCBI; Oar_4.0). Differentially expressed genes (DEGs) were identified as having a FDR ≤ 0.05, ≥ 2-fold change, and ≥ 0.2 RPKM. The number of DEGs that were up (gray) or down (black)-regulated is indicated based on obesity status, sex, and changes that were unique in individual comparisons between obese/lean versus male/female conceptuses

There were 21 differentially expressed genes (DEGs) in conceptuses collected from lean versus obese (see Additional file 2). Of these, 10 DEGs were down-regulated and 11 were up-regulated in conceptuses derived from obese ewes (Fig. 1; Table 3). Analysis with the PANTHER GO-Slim Biological Process system identified cellular process (GO: 0009987), metabolic process (GO: 0008152), and cellular component organization (GO: 0071840) as the three largest GO categories represented in the DEGs (11, 6 and 3 genes respectively). KEGG pathway analysis was also performed to analyze the various biological pathways represented within the DEG list. KEGG pathway analysis identified DEGs involved in the PI3K-AKT signaling pathway (*PPP2R3A* and *BRCA1*), with specific involvement cell proliferation, angiogenesis and DNA repair. Also, 4 of these DEGs have a known-role in placenta development and function (*ALCAM, BRCA1 GP2, GSTA4)* and 5 are associated with obesity and insulin resistance (*MPHOSPH9, BRCA1, ASP, ALCAM, GP2*).

**Table 3:** Obese versus lean DEGs identified in conceptus samples and associated gene ontology (GO) terms

Gene ID	Fold change, obese vs lean (Log_2_)^a^	Cellular process	Metabolic process	Cellular component organization	Proliferation, angiogenesis, DNA repair	Obesity and insulin resistance	Placenta^b^
*MPHOSPH9*	8.66					**+**	
*INTS12*	8.46						
*CEP57L1*	8.41						
*BRCA1*	7.44	**+**	**+**	**+**	**+**	**+**	**+**
*ASP*	6.65	**+**	**+**			**+**	
*PAPD4*	6.53	**+**					
*ALCAM*	6.31	**+**				**+**	**+**
*RAB4B*	5.94						
*TTK*	5.26	**+**	**+**				
*RPS3A*	3.66	**+**	**+**				
*TUBA3E*	1.62	**+**		**+**			
*PPP2R3A*	−4.42	**+**	**+**		**+**		
*GSTA4*	−4.78						**+**
*GP2*	−5.55					**+**	**+**
*DIS3L2*	−6.0						
*FAM213A*	−7.12		**+**				
*SLC35B3*	−7.66	**+**					
*PAAF1*	−8.04						
*ERMARD*	−8.24						
*DYNLL2*	−8.61	**+**					
*TPM1*	−8.89	**+**		**+**			

^a^relative fold change (FDR ≤ 0.05, ≥ 2-fold change, ≥ 0.2 RPKM)

^b^The term “Placenta” is not an official GO term. It represents genes identified through a literature search using the search terms “placenta”, “trophectoderm”, and “trophoblast”

“+” indicates the presence of the DEG within the specific GO term

### Conceptus sex-dependent changes in gene expression

Conceptus sex also affected transcript profiles. A total of 137 DEGs (109 annotated, 28 unannotated) were detected between male and female conceptuses (see Additional file 3). Of these, 25 DEGs were down-regulated and 112 were up-regulated in male vs female conceptuses (Fig. 1). Gene ontology terms associated with the DEGs include primary metabolic processes, regulation of biological processes, cell death and transport (23, 18, 4, and 8 DEGs, respectively) (Table 4). KEGG analysis identified 9 DEGs involved in metabolic processes (*ALDH1A1, B3GALNT1, CKB, CKM, DDC, HPSE, ISYNA1, NMRK, PMM*). Metabolic processes represented include glycan biosynthesis and metabolism, carbohydrate metabolism, amino acid metabolism, and the metabolism of cofactors and vitamins, specifically nicotinate and nicotinamide. KEGG analysis also identified protein digestion and absorption (*MME, PAG11, PAG4, PAG9*), and arginine and proline metabolism (*CKB, CKM*) to be affected by conceptus sex. Lastly, 33 of these DEGs have a reported involvement in placental development and function.

**Table 4:** Biological terms detected based on conceptus sex

Biological GO term	Greatest differential expression	# DEGs^a^	% total DEGs
Primary metabolic processes	*DENND4C, ENTPD1,* *GUCY2C, PAG4, PAG9*	23	16.7
Regulation of biological processes	*CASP6, DKK4,* *FGFR1, GUCY2C, SS18*	18	13.2
Cell death	*ADAM19, BCL2A1,* *CASP6, FGFR1*	4	2.9
Transport	*PMM2, RAB31,* *SLC25A12, SNX16, XPO4*	8	5.8
Placenta^b^	*CPA4, PAG4, TPM1, IL2RB, PRP4,*	33	24.1

^a^FDR ≤ 0.05, ≥ 2-fold-change, ≥ 0.2 RPKM

^b^The term “Placenta” is not an official GO term. It represents genes identified through a literature search using the search terms “placenta”, “trophectoderm”, and “trophoblast”

### Maternal obesity differentially impacted gene expression in each conceptus sex

There were 347 DEGs detected when comparing differences in how each conceptus sex was affected by exposure to obese and lean maternal conditions (see Additional file 4).

**Fig. 2:**
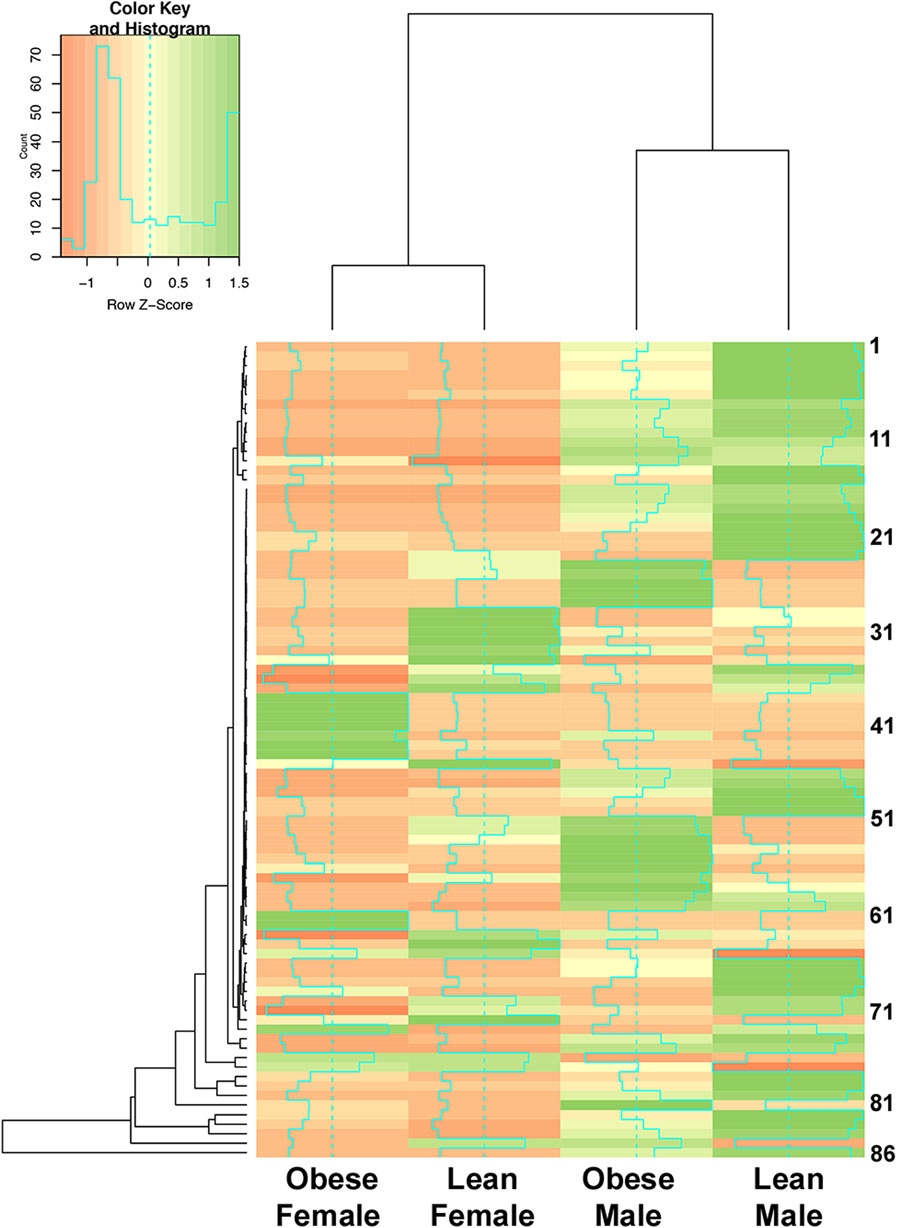
Heat map showing clustering patterns for placenta-associated DEGs exhibiting sex and obesity-dependent effects. Ovine conceptuses were collected from obese and lean ewes on day 14 of pregnancy by uterine flush. Conceptus sex was determined by PCR using X- and Y-specific primers, and 4 conceptuses/sex/treatment underwent RNA sequencing (N = 16 total conceptuses). Sequencing analysis was performed using Genomics Workbench 10.1.1 (CLC bio). Sequences were mapped to the *Ovis aries* genome (NCBI; Oar_4.0), and differentially expressed genes (DEGs) were identified as having a FDR ≤ 0.05, ≥ 2-fold change, and ≥ 0.2 RPKM. Genes involved in placentation were identified from the list of DEGs via literature search. Individual gene IDs can be found in Additional file 6

Between 23 and 167 DEGs were identified in the specific pair-wise comparisons (Fig. 1). The largest numbers of DEGs were detected for lean-derived male versus lean-derived female conceptuses and lean-derived males versus obese-derived females. When examining all the various DEGs as one dataset, 86 of the DEGs were involved in placenta development and function. These DEGs, including several instances where DEGs contained multiple gene variants, are organized on a heat map to describe differential expression trends between the various maternal obesity and conceptus sex groups (Fig. 2, Additional file 5). These DEGs segregated initially based on conceptus sex and thereafter based on obesity status.

## Discussion

Human obesity rates continue to climb in the United States. Though predominantly attributed to lifestyle choices, recent findings suggest exposure to maternal obesity in utero can produce similar metabolic and physiological outcomes in offspring regardless of their postnatal diet [33]. Studies utilizing the mouse model have identified changes in development following obesity exposure during the earliest stages of development [34, 35]; however, an understanding of the timing of these events is currently lacking in sheep. Work until now has focused on characterizing fetal and postnatal outcomes of maternal obesity [12, 13, 31, 32]. The obese ewe produces offspring that exhibit altered growth, adiposity, and glucose tolerance in adulthood [13]. However, the specific times during development when obesity can impact embryonic and fetal programming remained unexplored.

This work sought to establish whether programming events resulting from obesity could be detected early in pregnancy, and specifically during the peri-implantation period. This allowed us to examine changes in gene expression that would occur solely from alterations in oocyte maturation, fertilization, and embryonic and conceptus development. The extended period of pre-implantation conceptus development that occurs in the sheep and other ruminants permitted us to collect large conceptus samples that were at least largely and potentially totally devoid of endometrium [36, 37]. Collecting at this time also provided us with the opportunity to examine conceptuses when they were comprised primarily of extraembryonic membranes, and specifically trophectoderm and endoderm. This permitted a detailed description of how obesity status impacts early placental development and allowed us to identify the existence of early developmental programming in the sheep.

One interesting facet of this study was that many of the metabolic and endocrine parameters normally associated with obesity in humans and rodents were not evident in this work. Notably, NEFA concentrations were not affected by obesity status. This opposes the findings of previous studies that report an increased plasma NEFA concentration accompanying the obese phenotype of sheep [38, 39]. However, this same NEFA response has been reported in pregnant rats maintained on high fat diets [40]. We propose that the increase in NEFA concentrations in obese and lean ewes at D0 occurred because ewes were in estrus, where mating usually will take precedence over eating. Likewise, glucose concentrations were unaffected by obesity status in this study. This is not surprising given that ruminants utilize volatile fatty acids for a constant-state level of glucose production, whereas monogastrics actively absorb glucose. This means an obese state was achieved in this work without inducing hyperglycemic or diabetic states.

Obesity can negatively impact the establishment of pregnancy in cow and human models [41, 42]. However, previous work in the sheep reported no effect of donor ewe adiposity on ovulation rate, fertilization rate, pregnancy rate, conceptus growth, or birth weight. [31, 43]. It was interesting to observe that obesity-dependent changes in peri-implantation conceptus gene expression exist in the absence of effects on ovulation rate, pregnancy rate, pregnancies per ovulation, conceptus length, P4 production, and IFNT production. A small group of DEGs existed (*n* = 21). Several DEGs were part of various GO terms involving cellular and metabolic processes and cellular component organization. These findings are supported by studies in the rodent model, which describe reduced blastocyst rates, retarded embryonic development, and altered regulation of crucial metabolic genes following exposure to maternal obesity [44, 45].

Some of these obesity-dependent changes in gene expression noted in this work may represent early signs of adiposity in offspring [46]. Five of the twenty-one obesity-dependent DEGs are associated with obesity and insulin resistance (*MPHOSPH9, BRCA1, ASP, ALCAM, GP2*) [47–51]. Also, DEGs associated with placental development were detected in this work. Placental mal-programming is associated with various peri- and post-natal disorders, and at least two obesity-dependent DEGs have been associated with placental disorders. The first is activated leukocyte cell adhesion molecule (*ALCAM*), which is a TE-expressed protein in human placentae whose expression is diminished during preeclampsia [52, 53]. The second is breast cancer gene 1 (*BRCA1*), a tumor suppressor protein that facilitates DNA repair or cell destruction after DNA damage. This factor also plays active roles in TE proliferation and invasion [54–56].

Conceptuses exposed to maternal obesity also showed differential expression of genes associated with response to oxidative stress (*BRCA1, GSTA4*). Oxidative stress occurs naturally in the uterus, and oxidation is an essential facet of embryogenesis [57]. Oxidative stress may also impair development with decreases in embryonic competency and cell survival observed in stressed versus non-stressed embryos [58, 59]. These previous findings compliment the DEGs associated with DNA repair identified in this work. The differential expression of genes involved in the response to oxidative stress may indicate abnormal oxygen environment in utero, thus resulting in changes in gene expression in conceptuses exposed to maternal obesity.

It also was important that sex of the conceptus be considered in this work. There are numerous examples in mammals of how maternal obesity and other intrauterine stresses differentially affect male and female fetuses (reviewed in [60, 61]). The mechanisms behind the differential developmental programming of male and female embryos is not completely known. In early embryogenesis this likely occurs, at least in part, by incomplete X-inactivation in female conceptuses, leading to an up-regulation of X-linked genes in female conceptuses. In cattle, X-inactivation occurs primarily between the blastocyst stage and day 14 of conceptus development, although, sexual dimorphism still exists by day 19 of pregnancy in this species [62, 63]. There is no evidence suggesting that ovine conceptuses experience a similar ontogeny of X-linked gene inactivation, and certainly the timing of these events will be shifted forward by 3 to 4 days given the more rapid development of ovine conceptuses prior to implantation [64]. However, the closeness between these species in early conceptus development make this phenomenon an attractive explanation for at least some of the sex-dependent events detected in this work. Additionally, it is possible that male and female embryos respond differently to uterine histotroph during early embryogenesis. This idea is reinforced by studies reporting sexually dimorphic gene expression as early as the morula and blastocyst stages in cattle [65, 66]. This variation in gene expression may lead to disparities in cell survival and lineage specification during early development in male and female embryos.

One set of sex-dependent DEGs of special note are the placental-specific aspartic proteases that are known as pregnancy-specific glycoproteins (PAGs). In ruminants, PAGs are classified as ancient or modern members of this multigenic family based on whether they are produced solely from mononucleated or binucleated TE, respectively [67]. Five PAG gene transcripts were identified herein (*PAG1, 2, 4, 9, 11*), which represent both modern and ancient categories. In all instances, these PAG transcripts were greater in abundance in male conceptuses than female conceptuses. The greatest difference was *PAG9*, which was 220-fold greater in male than female conceptuses. Individual PAG genes are expressed at different stage of pregnancy in cattle and presumably other ruminants. Several of the PAGs identified here are expressed early in gestation (e.g. *PAG4, 9*) whereas the others are produced throughout gestation but predominantly during mid- and late-gestation [68, 69]. Identifying PAGs produced by both TE cell types indicates that this outcome does not reflect changes in the distribution of mononucleate and binucleate cell types. The biological significance of PAGs remain unclear.

Several other notable DEGs were identified based on conceptus sex. Fibroblast growth factor receptor 1 (*FGFR1*) is one of four tyrosine kinase receptor genes that control the various actions of FGFs throughout the body [70]. It is implicated as a contributing factor to fetal growth restriction induced by placental insufficiency in women [71, 72]. Another is mucin-15 *(MUC15*), a cell membrane-bound mucin that controls TE invasion [73, 74]. A third DEG of note is the amino acid transporter, *SLC6A14*, is a sodium-dependent, neutral and cationic amino acid transporter. Though it has a broad specificity, *SLC6A14* is essential for leucine uptake in mouse TE [75]. Also, a disintegrin and metalloprotease 19 (*ADAM19*) impacts TE invasion and adhesion in the human [76]. Lastly, placental lactogen and one member of the placental prolactin-related protein (PRP) family (termed *PRP4*) was differentially expressed in male and female conceptuses. Collectively, the differential expression of these genes implies that there is a naturally-occurring sexual dimorphism in placental development and function.

Exposure to maternal obesity affected male and female conceptuses differently. The individual comparisons generally did not share DEGs, but the same GO terms with the highest DEG representation were similar in both male and female conceptuses. These were cellular processes and metabolic processes. Work in the area of developmental programming utilizing mouse, rat and ovine models shows that male and female offspring respond differently in the presence of various environmental stressors in utero [21, 77, 78], and a similar phenomenon appears to be present in this model.

Samples were collected at day 14 of gestation, during the elongation phase of conceptus development and just prior to implantation into the uterus [37]. This phase of development is marked by an exponential increase in length of the trophectoderm, with the conceptus growing from 1 mm on day 11 to around 19 cm on day 15 [79, 80]. At the time of collection, the trophectoderm is the predominant tissue of the conceptus. Thus, it is not surprising that a subset of DEGs were associated with placental development and function. Between 19 and 26% of the obesity, sex, and obesity by sex DEGs were related to the placenta. An official GO term is not available for placental development and function, so this DEG category was developed in-house by identifying DEGs that have been studied in the placenta, trophectoderm and/or trophoblast of humans, rodents and/or domestic animals. The DEGs identified through this search included those that contained various placental functions, including trophoblast adhesion and implantation, placental vasculature and angiogenesis, and cellular responses to hypoxia and preeclampsia. Although a majority of the published reports used for this assessment were made in post-implantation or late gestation placentae (for examples, see [19, 81, 82]), there were several noteworthy outcomes that relate to post-implantation and late gestational problems. Abnormal TE adhesion and implantation are recognized precursors to preeclampsia in humans, as preeclampsia is characterized by shallow TE invasion [83, 84]. Likewise, pro- and anti-angiogenic factors are misregulated in preeclampsia, resulting in hypertension, the clinical hallmark of preeclampsia [85]. Therefore, while samples in this study were collected immediately prior to uterine implantation, it appears that the mechanisms responsible for implantation and placentation are already perturbed at day 14 of gestation. Furthermore, these maladaptive placental precursors may help to explain the altered gestational growth trajectory observed in ewes born to obese ewes [46].

It remains unclear what is driving these obesity-dependent changes in conceptus gene expression. It certainly is possible that direct interactions of maternally-derived factors (e.g. hormones, metabolites) may be promoting changes in conceptus gene expression, although we have not identified any indication of this type of regulation in the gene ontology screening. A more probable explanation is that the uterus is driving these conceptus responses to maternal obesity. Uterine secretions (i.e. histotroph) control peri-implantation conceptus development in sheep and other ruminants [86]. Progesterone is a central controller of histotroph production in early pregnancy [87], but progesterone concentrations were not affected by obesity status in this study. An alternative way uterine function may be influenced is by low-grade inflammatory events that accompany obesity [88]. Localized inflammatory responses are observed in the rat and horse uterus in an obese state, and these responses are probably driven, at least in part, by pro-inflammatory cytokine actions within the endometrium [89, 90]. These and other pro-inflammatory factors likely affect uterine homeostasis in ways that cause conceptuses to respond differently to their uterine environment.

## Conclusions

These results indicate that the conceptus genome is susceptible to perturbations caused by maternal obesity early in development, even though morphological changes to the conceptus nor alterations in maternal reproductive parameters are detectable. These effects of maternal obesity also are sexually dimorphic. Furthermore, this work identifies genes involved with placental development, and specifically adhesion, implantation, angiogenesis and placental vasculature as major targets of genetic regulation. The altered expression of these transcripts may be some of the earliest indications of implantation failure and subsequent placental insufficiency that are observed in obese females. Further work should focus on identifying the morphological changes resulting from the misregulation of these placental genes in later gestation.

## Methods

### Animal use

Sheep used in this work were provided by the Virginia Tech Sheep Center (Blacksburg, VA). All animal work was completed in compliance and with the approval of the Virginia Tech Institutional Animal Care and Use Committee (IACUC; #14–104).

Dietary treatments were imposed ~ 4 months prior to the start of the study to establish the obese and lean phenotypes. Dorset ewes, 1–3 years in age, were assigned randomly to lean or obese groups. The obese state was induced by feeding 1 kg corn/day and providing ad libitum exposure to high quality pasture in the summer and orchard grass hay in the fall and winter months. Ewes that achieved a body condition score (BCS) ≥4 (scale of 1–5) were considered “obese” as per the BCS standards described by Thompson and Meyer [91]. Lean ewes were kept on a maintenance diet composed of previously grazed pasture in the summer months and poor-quality hay in the fall and winter months. Ewes with a BCS of 2.5–3 where chosen from this group. Back fat measurements were collected on a subset of ewes (*n* = 4 lean and 5 obese ewes) via ultrasonography. Once an obese and lean ewe model was established, animals were subjected to an estrous synchronization protocol in fall and winter months (September to February). The protocol began with controlled internal drug release (CIDR) device (Pfizer, New York, NY) insertion and Cystorelin (Merial, Lyon, France) injection (50 μg; IM) followed 7 days later with CIDR removal and Lutalyse (Zoetis, Parsippany, NJ) injection (15 mg; IM) [92]. Ewes were then bred to genetically-related Dorset rams (three-quarter siblings).

### Blood analyses

Blood samples were collected from the jugular vein at day 0, 6, and 14 of gestation (day 0 = day of breeding) and maintained on ice until plasma was isolated via centrifugation (1500 g × 15 min). Plasma was stored at − 20 °C. Ewes were kept off-feed for 12 h prior to the day 14 blood collections. Plasma NEFA concentrations were determined using the NEFA-HR(2) Microtiter procedure according to manufacturer instructions (Wako Diagnostics, Mountain View, CA). Plasma progesterone concentrations were determined using the IMMULITE 2000 XPi Immunoassay system (Siemens Medical Solutions Diagnostics, Tarrytown, NY state). Plasma glucose concentrations were assessed using Glucose Colorimetric Assay Kit (Ann Arbor, MI).

### Conceptus collections

Ewes were sacrificed on day 14 of gestation. Body weight was recorded at the time of sacrifice. The uterus was excised by mid-ventral dissection. Each uterine horn was flushed with 30 mL Dulbecco’s PBS [pH 7.2] (Gibco, Gaithersburg, MD) to recover conceptuses. Individual conceptuses were teased apart, and each conceptus length was recorded. An example of a flushed conceptus is shown in Additional File 6. The number of corpora lutea (CL) was recorded and used to determine the percentage of pregnancies per ovulation. Individual conceptuses were snap-frozen in liquid nitrogen, and stored at − 80 °C.

### IFNT analysis

Individual uterine flushes were assessed for interferon-tau (IFNT) protein content by the ISRE-Luc bioassay described previously by this laboratory [93]. In brief, Madin-Darby bovine kidney cells (MDBK; ATCC#CCL-22) that were transduced with an ISRE-Luc reporter were plated into 96-well polystyrene plates with opaque walls and optically clear bottoms (Corning Inc., Corning, NY) at a density of 5–10 × 10^5^ cells/well in Dulbecco’s modified eagle medium (DMEM, 25 mM glucose; Life Technologies, Grand Island, NY) containing 10% (*v*/v) fetal bovine serum (FBS), and antibiotics (50 IU Penicillin G and 50 μg/ml Streptomycin sulfate). After 4 h incubation at 37 °C in 5% CO_2_, medium was replaced with 50 μl of medium and either the sample or standard. Recombinant human IFNA was used as the assay standard (3.87 × 10^8^ IU/mg; EMD Biosciences, Billerica, MA). A 1:3 serial dilution of IFNA was completed to generate the standard curve. Samples were prepared by mixing DMEM containing 10% FBS and antibiotic with the flush solution (no more than one-half the final volume of medium added to each well). Cells were incubated at 37 °C overnight (16–24 h). Luciferase activity was determined by adding 50 μl of One-Glo Luciferase Assay Substrate (Promega Corp., Madison, WI) to each well. After 10 min of agitation, the plate was read using an Infinite M200 PRO Plate Reader (TECAN Systems Inc., San Jose, CA).

### RNA and DNA extraction

Conceptus RNA and DNA were isolated using the AllPrep DNA/RNA mini kit (Qiagen, Hilden, Germany). Prior to PCR analysis, samples underwent an on-column DNase1 digestion (Life Technologies, Carlsbad, CA). Samples were reverse transcribed using a High Capacity cDNA Reverse Transcription Kit (Life Technologies). Quality of RNA was examined using the Experion RNA StdSens Analysis Kit (BioRad, Hercules, CA).

### Conceptus sexing

Conceptus sex was determined using a previously described PCR-based approach [94] using GoTaq Green Master Mix (Promega, city state) and an Eppendorf Realplex4 Mastercycler (Hamburg, Germany). The thermocycler was programmed for an initial 5 min, 95 °C denaturation step followed by 40 cycles of 95C, 56 °C. and 72 °C, and ending with a 5-min polishing step at 72 °C. Samples were then digested with the Sac1 enzyme for 3 h at 37 °C, loaded onto a 1% (*w*/*v*) agarose gel and electrophoresed. DNA was detected using SYBR Safe DNA gel stain (ThermoFisher, Waltham, MA). Male conceptuses were identified by the presence of 3 bands, while females appeared as a double band.

### RNA-sequencing analysis

RNA samples (*n* = 4 samples/sex/treatment; 16 total samples) were sequenced by Cofactor Genomics (St. Louis, MO), using an Illumina-based sequencing platform using single end 75 base reads.

Sequencing analysis was performed using CLC Genomics Workbench 10.1.1 (Qiagen; Germantown, MD). Reads were imported in CLC genomics workbench and cleaned to remove reads containing adapters and low-quality reads from raw data. Sequences were then aligned to the *Ovis aries* reference genome (NCBI; Oar_4.0) from Ensembl. Sequences were also mapped to the *Bos taurus* (Ensembl;UMB3.1) and *Capra hircus* (NCBI; ASM170441v1) genomes for an initial comparative analysis. Expression values were expressed in reads per kilobase of transcript per million (RPKM). An empirical analysis of differential gene expression was performed using the Robinson and Smyth Exact Test (Robinson and Smyth, 2007). A negative binomial distribution (NB) was assumed. False discovery rate (FDR) was controlled at a rate of 5% using the Benjamini-Hochberg method (Benjamini and Hochberg, 1995). The list of DEGs also were limited to those containing ≥2-fold change and ≥ 0.2 RPKM. Gene ontology (GO) groupings were examined in DEGs using the functional classification analysis in the PANTHER Classification System (version 12.0). KEGG Mapper (v3.1) was used for DEG pathway analysis. Placenta-associated genes were identified through a literature search using the search terms “placenta”, “trophectoderm”, and “trophoblast” as there are not currently GO categories for these terms.

### Statistical analysis

Ewe body weight, metabolic parameters and reproductive parameters were analyzed using the general linear model of the statistical analysis system (SAS Institute, Cary, NC). Conceptus sex ratio was analyzed using PROC FREQ of SAS. A repeated measures analysis and within day ANOVA were used to analyze plasma NEFA, glucose and progesterone data (SAS Institute, Cary, NC).

## Additional files

Additional file 1: Raw data containing all transcripts identified in the RNA-sequencing analysis. Included are reference sequence (RefSeq) and gene ID for each transcript, the max group mean (indicating the mean RPKM for the treatment group with the greatest expression value within each comparison), log_2_ fold-changes based on obesity status, sex and (XLSX 17599 kb)

Additional file 2: Obese-derived vs Lean-derived conceptus DEGs. Sequences were mapped to the *Ovis aries* genome (NCBI; Oar_4.0), and differentially expressed genes (DEGs) were identified as having a FDR ≤ 0.05, ≥ 2-fold change, and ≥ 0.2 RPKM. (XLSX 11 kb)

Additional file 3: Male vs Female conceptus DEGs. Sequences were mapped to the *Ovis aries* genome (NCBI; Oar_4.0). Differentially expressed genes (DEGs) were identified as having a FDR ≤ 0.05, ≥ 2-fold change, and ≥ 0.2 RPKM. (XLSX 23 kb)

Additional file 4: DEGs that differed each conceptus sex based on maternal obesity status. Sequences were mapped to the *Ovis aries* genome (NCBI; Oar_4.0). Differentially expressed genes (DEGs) were identified based on differences in conceptus sex in the obese and lean treatment groups (FDR ≤ 0.05, ≥ 2-fold change, and ≥ 0.2 RPKM). This table shows differences as they exist within the individual pair-wise comparisons for these various interacting effects, and specifically: 1) obese vs. lean male conceptuses, 2) obese vs. lean female conceptuses, 3) obese male vs. female conceptuses, 4) obese male vs. lean female conceptuses, 5) lean male vs. obese female conceptuses, and 6) lean male vs. female conceptuses. (XLSX 108 kb)

Additional file 5: Gene IDs for placenta-associated DEGs represented in Fig. 2**.** Sequences were mapped to the *Ovis aries* genome (NCBI; Oar_4.0), and differentially expressed genes (DEGs) were identified as having a FDR ≤ 0.05, ≥ 2-fold change, and ≥ 0.2 RPKM. Genes involved in placentation were identified from the list of DEGs via literature search. (XLSX 14 kb)

Additional file 6: Example of a uterine flush at day 14 breeding containing multiple conceptuses. The photograph was taken immediately after flushing the conceptus from the uterus. The conceptuses are intertwined at this time. After conceptuses are gently uncoiled, the length of each can be determined using the grided plates (1 cm grid). (TIF 212 kb)

## Abbreviations

ADAM19: ADAM Metallopeptidase Domain 19
ALCAM: Activated leukocyte cell adhesion molecule
ALDH1A1: Aldehyde Dehydrogenase 1 Family Member A1
ASP: Acylation-stimulating protein
B3GALNT1: Beta-1,3-N-Acetylgalactosaminyltransferase 1
BCS: Body condition score
BRCA1: Breast cancer 1
CIDR: Controlled internal drug release
CKB: Creatine Kinase B
CKM: Creatine Kinase, M-Type
CL: Corpus luteum
DDC: Dopa Decarboxylase
DEG(s): Differentially expressed gene(s)
DMEM: Dulbecco’s Modified Eagle Medium
DNA: Deoxyribonucleic acid
DOHAD: Developmental origins of health and disease
FBS: Fetal bovine serum
FDR: False discovery rate
FGFR1: Fibroblast growth factor receptor 1
FOAD: Fetal origins of adult disease
GO: Gene ontology
GP2: Glycoprotein 2
GSTA4: Glutathione S-Transferase Alpha 4
HPSE: Heparanase
IFNA: Interferon-alpha
IFNT: Interferon-tau
ISRE: Interferon stimulated response element
ISYNA1: Inositol-3-Phosphate Synthase 1
MME: Membrane Metalloendopeptidase
MPHOSPH9: M-Phase Phosphoprotein 9
MUC15: Mucin-15
NCBI: National Center for Biotechnology Information
NEFA: Non-esterified fatty acids
NMRK: Nicotinamide riboside kinase
P4: Progesterone
PAG11: Pregnancy associated glycoprotein 11
PAG2: Pregnancy associated glycoprotein 2
PAG4: Pregnancy associated glycoprotein 4
PAG9: Pregnancy associated glycoprotein 9
PCR: Polymerase chain reaction
PMM: Phosphomannomutase
PPP2R3A: Serine/threonine-protein phosphatase 2A regulatory subunit B″ subunit alpha
PRP4: Prolactin-related protein family 4
RNA: Ribonucleic acid
RPKM: Reads per kilobase million
SLC6A14: Solute Carrier Family 6 Member 14
TE: Trophoblast
