# Supplementary figures and images for: Exposure to maternal obesity alters gene expression in the preimplantation ovine conceptus
Source: BMC Genomics. 2018 Oct 11;19:737. doi: 10.1186/s12864-018-5120-0 (PMC6180665; doi:10.1186/s12864-018-5120-0)

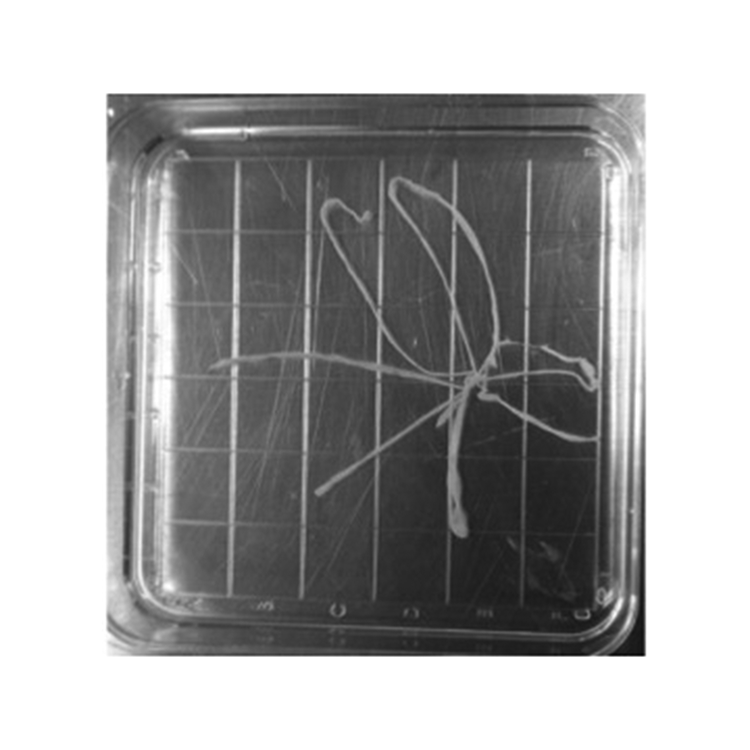

Supplement: Supplementary file 6 — Example of a uterine flush at day 14 breeding containing multiple conceptuses. The photograph was taken immediately after flushing the conceptus from the uterus. The conceptuses are intertwined at this time. After conceptuses are gently uncoiled, the length of each can be determined using the grided plates (1 cm grid). (TIF 212 kb) [file 12864_2018_5120_MOESM6_ESM.tif]
